# The consistencies and inconsistencies between distal cholangiocarcinoma and pancreatic ductal adenocarcinoma: A systematic review and meta-analysis

**DOI:** 10.3389/fonc.2022.1042493

**Published:** 2022-12-12

**Authors:** Tian-Run Lv, Ju-Mei Wang, Wen-Jie Ma, Ya-Fei Hu, Yu-Shi Dai, Yan-Wen Jin, Fu-Yu Li

**Affiliations:** Department of Biliary Surgery, West China Hospital, Sichuan University, Chengdu, Sichuan, China

**Keywords:** pancreatic ductal adenocarcinoma, pancreaticoduodenectomy, prognosis, distal cholangiocarcinoma, ampullary cancer

## Abstract

**Objective:**

To evaluate the consistencies and inconsistencies between distal cholangiocarcinoma (DCCA) and pancreatic ductal adenocarcinoma (PDCA) regarding their biological features and long-term prognosis.

**Methods:**

PubMed, the Cochrane Library, and EMBASE were searched to find comparative studies between DCCA and PDCA. RevMan5.3 and Stata 13.0 software were used for the statistical analyses.

**Results:**

Eleven studies with 4,698 patients with DCCA and 100,629 patients with PDCA were identified. Pooled results indicated that patients with DCCA had a significantly higher rate of preoperative jaundice (p = 0.0003). Lymphatic metastasis (p < 0.00001), vascular invasion (p < 0.0001), and peri-neural invasion (p = 0.005) were more frequently detected in patients with PDCA. After curative pancreaticoduodenectomy (PD), a significantly higher R0 rate (p < 0.0001) and significantly smaller tumor size (p < 0.00001) were detected in patients with DCCA. Patients with DCCA had a more favorable overall survival (OS) (p < 0.00001) and disease-free survival (DFS) (p = 0.005) than patients with PDCA. However, postoperative morbidities (p = 0.02), especially postoperative pancreatic fistula (POPF) (p < 0.00001), more frequently occurred in DCCA.

**Conclusion:**

Patients with DCCA had more favorable tumor pathological features and long-term prognosis than patients with PDCA. An early diagnosis more frequently occurred in patients with DCCA. However, postoperative complications, especially POPF, were more frequently observed in patients with DCCA.

## Introduction

Distal cholangiocarcinoma (DCCA) and pancreatic ductal adenocarcinoma (PDCA) are both defined as peri-ampullary cancers, sharing incidences of 20% and 70%, respectively (1–3). Curative resection provides the only chance of curing these deadly malignancies, and pancreaticoduodenectomy (PD) has been widely applied in patients with DCCA and PDCA. However, although these two rare entities share similar surgical procedures and have a relatively indistinguishable tumor sites, whether they can be treated equally remains controversial.

Macroscopically, DCCA arises from the epithelium of the distal bile duct and often involves posterior pancreatic margins, while PDCA arises from the pancreatic ducts and can occur in any part of the pancreas (4). There are no specific tumor biomarkers to date to distinguish them clearly (4). Moreover, accumulating evidence has suggested that despite their similar origin and surgical techniques, their clinical-pathological features and long-term prognosis were reported to be inconsistent to some extent (4–9). For example, Andrianello et al. revealed that lymphatic invasion and neural invasion were more frequently detected in patients with PDCA, and patients with DCCA had a significantly better prognosis (5). However, the study by Guilbaud et al. revealed that both tumor types shared equal prognosis and oncological outcomes (8). The study introduced above either included a small sample size or just evaluated their differences from a few tumor-related features. The small sample size and the inadequate parameters continued undermining the validity of their results and conclusions. Obviously, powerful evidence is lacking, and our meta-analysis is performed to have a more comprehensive evaluation on their similarities and differences.

## Materials and methods

### Search strategy

The Preferred Reporting Items for Systematic Reviews and Meta-Analyses (PRISMA) statements were the major guidelines observed (10). PubMed, EMBASE, and the Cochrane Library were searched until 1 May 2022. Eligible studies were restricted to comparative studies between DCCA and PDCA. The following keywords were used: distal cholangiocarcinoma, pancreatic ductal carcinoma, extra-hepatic cholangiocarcinoma, ampullary cancer, bile duct stenosis, and prognosis. Other relevant studies were also screened.

### Inclusion and exclusion criteria

The following were the inclusion criteria: 1) comparative studies between DCCA and PDCA or various ampullary cancers, 2) studies that reported the tumor’s clinical-pathological features or oncological outcomes or long-term survival, and 3) published English articles. Abstracts, meetings, letters, reviews, or comments as well as studies that shared completely the same database were ruled out.

### Quality assessment and statistical analyses

The specific modalities within our manuscript regarding the quality evaluation of identified studies and statistical analyses are similar to those of our previous series (11). In order to reduce the similarity index, no illustrations will be provided (**Table 1**).

**Table 1:** Baseline characteristics of all studies included.

Author	Study period	Patient source	No. patients		Tumor classification criteria (DCCA *vs.* PDCA)	Pathological evaluation criteria	Follow-up (months)	Quality score (NOS)
			DCCA	PDCA				
Geenen RCI et al., 2001	1992–1998	Academic Medical Center, Amsterdam, The Netherlands	32	108	Pathological examination and the location of the tumor epicenter	NA	Median 15	7
Riall TS et al., 2006	1970–1999	Johns Hopkins Hospital, USA	135	564	Pathological examination and the location of the tumor epicenter	NA	Median 97	6
Hatzaras I et al., 2010	1992–2007	The Ohio State University, Columbus, OH, USA	18	249	Pathological examination and the location of the tumor epicenter	NA	NA	6
He J et al., 2014	1980–2011	Johns Hopkins Hospital, USA	317	1688	Pathological examination and the location of the tumor epicenter	NA	NA	6
Gonzalez RS et al., 2016	NA	Emory University Hospital, USA	47	109	Pathological examination and the location of the tumor epicenter	AJCC 7th edition	Median 18, range (0–157)	6
Andrianello S et al., 2016	1998–2014	University of Verona Hospital Trust, Italy	54	656	WHO classification of tumors of the digestive system	AJCC 7th edition	Median 24, range (3–173)	8
Ethun CG et al., 2016	2000–2014	US Extra-hepatic Biliary Malignancy Consortium; Central Pancreas Consortium	224	1239	Pathological examination and the location of the tumor epicenter	AJCC 7th edition	Median 14.7, IQR (6.1–32.1)	8
Hester CA 2018	2004–2012	National Cancer Database	3732	95511	NA	AJCC 6/7th edition	NA	8
Garnier J et al., 2021	2010–2018	Institut Paoli-Calmettes, Marseille, France	67	288	Pathological examination and the location of the tumor epicenter	AJCC 7th edition	NA	7
Guilbaud T et al., 2021	2005–2017	Two tertiary referral centers, France	37	151	Preoperative imaging data, biopsy, and immunohistochemical analyses	AJCC 7th edition	Every 3 months during the first 2 years and every 6 months thereafter	8
Muttillo EM et al., 2021	2010–2018	Sapienza University, Italy	35	66	Postoperative histological examination	NA	NA	6

DCCA, distal cholangiocarcinoma; PDCA, pancreatic ductal adenocarcinoma; WHO, World Health Organization; AJCC, American Joint Committee on Cancer; IQR, interquartile range; NA, not available; NOS, Newcastle–Ottawa Quality Assessment Scale.

## Results

### Study identification and selection

**Figure 1:**
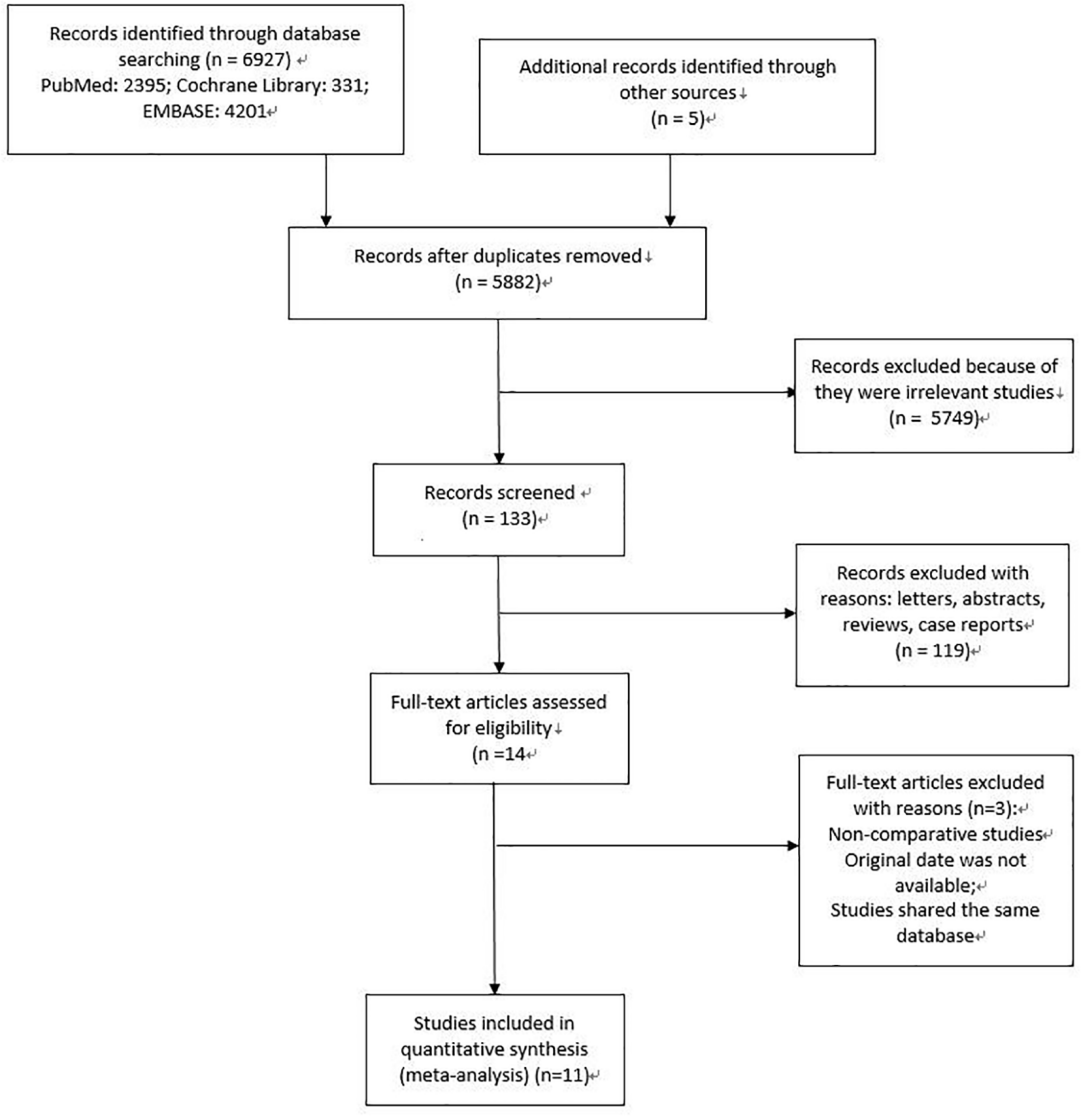
The specific process of literature research and selection.

A total of 6,932 studies were gained. Subsequently, under the inclusion and exclusion criteria, 11 studies were finally incorporated (**Figure 1**).

### Study characteristics

Initially, 12 studies (1, 4–9, 12–16) were identified through our search strategy. However, the study by Yeo et al. (16) (study period 1990–1996) and the study by He et al. (14) (study period 1980–2011) both came from Johns Hopkins Hospital. A complete overlap of the patient source was detected, and therefore, the study by Yeo et al. was excluded. Consequently, a total of 11 studies (1, 4–9, 12–15) with 4,698 patients with DCCA and 100,629 patients with PDCA were incorporated into our analysis. Except for the study by Garnier et al. (4), which was prospective, the remaining studies were retrospective cohort studies. All these studies reported the overall survival (OS) or disease-free survival (DFS) *via* the Kaplan–Meier curves. Nine studies also compared the clinical-pathological features between DCCA and PDCA (1, 4–8, 12–14). Additionally, the study by Hester et al. was based on the National Cancer Database (NCDB), and their patients were partly surgically treated and the others were undergoing palliative treatment (2004–2012) (7). Their study also analyzed the other two types of ampullary cancers, ampullary adenocarcinoma and duodenal adenocarcinoma, at the same time (7). The baseline characteristics of all studies are recorded in **Table 1**. There were a total of 13 measured parameters, including preoperative jaundice, preoperative stenting, R0 resection rate, lymph node metastasis, vascular invasion, neural invasion, tumor size, morbidities, postoperative biliary fistula (POBF), postoperative pancreatic fistula (POPF), mortalities, OS, and DFS. The pooled results of all available studies in measured outcomes are recorded in **Table 2**.

**Table 2:** Pooled results of all available studies in measured outcomes.

Outcomes	No. studies	No. patients		Model (fixed/random)	OR/HR/WMD	95% CI	p (overall test)	p^C^ (overall test)	Heterogeneity		Begg’s test		Egger’s test
		DCCA	PDCA						I^2^ (%)	p^H^	Pr > |z|*	Pr > |z|**	p > |t|*
Preoperative jaundice	4	141	1,164	Random	OR = 2.39	0.74–7.74	p = 0.15	p^C^ = 0.0003	70%	p^H^ = 0.02	0.497	0.734	0.343
Preoperative stenting	4	176	1,344	Random	OR = 0.95	0.20–4.48	p = 0.95	p^C^ = 0.95	91%	p^H^ < 0.00001	0.174	0.308	0.236
R0 resection rate	8	1,709	27,396	Random	OR = 1.93	1.25–2.97	p = 0.003	p^C^ < 0.0001	78%	p^H^ < 0.0001	0.138	0.174	0.128
Lymph node metastasis	8	2,111	35,422	Random	OR = 0.42	0.29–0.61	p < 0.00001	p^C^ < 0.00001	82%	p^H^ < 0.00001	0.048	0.063	0.207
Vascular invasion	6	695	3,723	Random	OR = 0.69	0.49–0.97	p = 0.03	p^C^ < 0.0001	59%	p^H^ = 0.03	0.188	0.260	0.265
Neural invasion	6	737	4,271	Random	OR = 0.47	0.28–0.79	p = 0.005	p^C^ = 0.005	85%	p^H^ < 0.00001	0.348	0.452	0.350
Tumor size	6	695	3,723	Random	WMD = −0.55	−0.72 to −–0.38	p < 0.00001	p^C^ < 0.00001	67%	p^H^ = 0.009	0.348	0.452	0.880
Morbidities	5	208	1,452	Random	OR = 1.43	0.77–2.64	p = 0.26	p^C^ = 0.02	71%	p^H^ = 0.009	0.327	0.462	0.520
POBF	3	158	1,095	Fixed	OR = 1.30	0.62–2.72	p = 0.49	p^C^ = 0.49	12%	p^H^ = 0.32	0.602	1	0.545
POPF ^(B-C)^	3	158	1,095	Fixed	OR = 3.52	2.46–5.06	p < 0.00001	p^C^ < 0.00001	14%	p^H^ = 0.31	0.602	1	0.625
Mortalities	5	208	1,452	Fixed	OR = 1.22	0.51–2.92	p = 0.65	p^C^ = 0.65	0%	p^H^ = 0.70	0.142	0.221	0.011
OS	9	4,485	99,016	Random	HR = 0.81	0.70–0.95	p = 0.009	p^C^ < 0.00001	74%	p^H^ = 0.0001	0.249	0.295	0.467
DFS	3	315	2,046	Random	HR = 1.20	0.68–2.12	p = 0.52	p^C^ = 0.005	83%	p^H^ = 0.0005	1	1	0.195

DCCA, distal cholangiocarcinoma; PDCA, pancreatic ductal adenocarcinoma; OR, odds ratio; HR, hazard ratio; WMD, weighted mean difference; CI, confidence interval; p^C^, corrected p-value; p^C^, corrected p-value after the sensitivity analysis; p^H^, p-value of the heterogeneity; POBF, postoperative biliary fistula; B-C, Clavien grade B to C; POPF, postoperative pancreatic fistula; OS, overall survival; DFS, disease-free survival.

*p-value; **p-value (continuity corrected).

#### Preoperative jaundice

**Figure 2:**
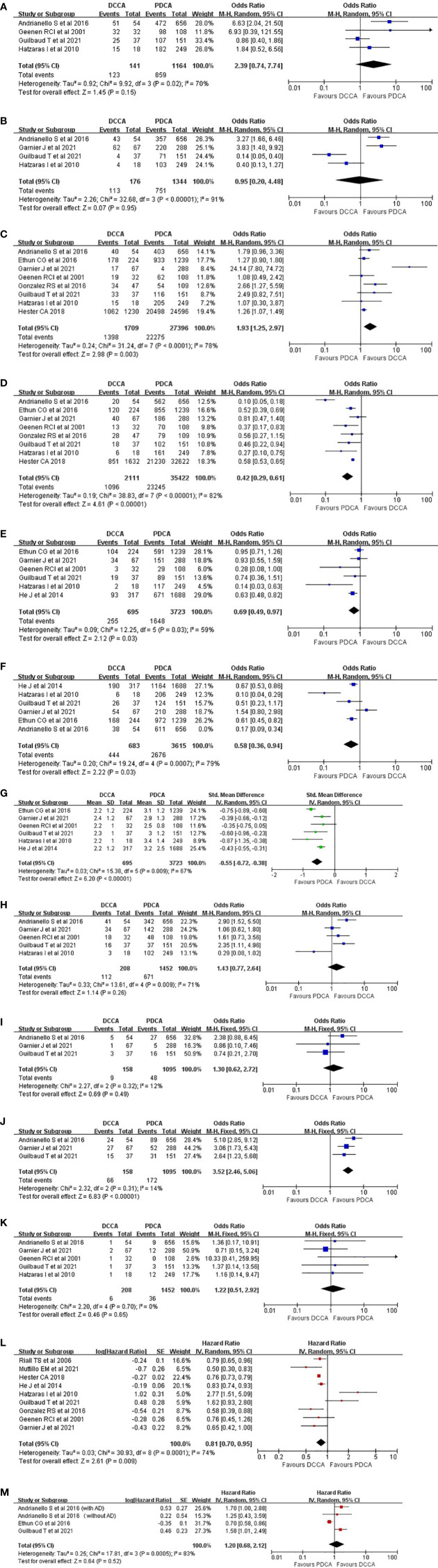
Pooled results regarding the tumor clinical-pathological features and long-term prognosis between DCCA and PDCA. **(A)** Preoperative jaundice. **(B)** Preoperative stenting. **(C)** R0 rate. **(D)** Lymph node metastasis. **(E)** Vascular invasion. **(F)** Neural invasion. **(G)** Tumor size. **(H)** Postoperative morbidities. **(I)** Postoperative biliary fistula. **(J)** Postoperative pancreatic fistula. **(K)** Mortalities. **(L)** Overall survival. **(M)** Disease-free survival. DCCA, distal cholangiocarcinoma; PDCA, pancreatic ductal adenocarcinoma.

Four studies (1, 5, 8, 12) regarding patients with preoperative jaundice were incorporated, and the pooled result revealed no difference (87.2% versus 73.8%, OR = 2.39, 95% CI 0.74 to 7.74; p = 0.15) (**Figure 2A**). Significant heterogeneity (χ^2^ = 9.92, p = 0.02, I^2^ = 70%) was detected, and when the study by Guilbaud et al. (8) was removed, low heterogeneity with a statistical difference was then detected (94.2% versus 74.2%, OR = 4.42, 95% CI 1.96 to 9.95; p = 0.0003) (χ^2^ = 2.37, p = 0.31, I^2^ = 16%).

#### Preoperative stenting

Four studies (1, 4, 5, 8) reported the number of patients who received preoperative stenting, and the pooled result revealed no significant difference (64.2% versus 55.9%, OR = 0.95, 95% CI 0.20 to 4.48; p = 0.95) (χ^2^ = 32.68, p < 0.00001, I^2^ = 91%) (**Figure 2B**).

#### R0 resection rate

Eight studies (1, 4–8, 12, 13) were incorporated, and a significantly higher R0 rate in patients with DCCA was acquired (81.8% versus 81.3%, OR = 1.93, 95% CI 1.25 to 2.97; p = 0.003) (χ^2^ = 31.24, p < 0.0001, I^2^ = 78%) (**Figure 2C**). When the study by Garnier et al. (4) was removed, low heterogeneity with a more significant p-value was acquired (84.1% versus 82.2%, OR = 1.33, 95% CI 1.16 to 1.53; p < 0.0001) (χ^2^ = 6.27, p = 0.39, I^2^ = 4%).

#### Lymph node metastasis

Eight studies (1, 4–8, 12, 13) were incorporated, and a significantly higher incidence of node metastasis in patients with PDCA was observed (51.9% versus 65.6%, OR = 0.42, 95% CI 0.29 to 0.61; p < 0.00001) (χ^2^ = 38.83, p < 0.00001, I^2^ = 82%) (**Figure 2D**). Heterogeneity analysis indicated that the study by Andrianello et al. (5) was the major source of heterogeneity.

#### Vascular invasion

Six studies (1, 4, 6, 8, 12, 14) were incorporated, and a significantly higher incidence of vascular invasion was acquired in patients with PDCA (36.7% versus 44.3%, OR = 0.69, 95% CI 0.49 to 0.97; p = 0.03) (χ^2^ = 12.25, p = 0.03, I^2^ = 59%) (**Figure 2E**). Heterogeneity analysis indicated that the study by Ethun et al. (6) was the major source of heterogeneity (32.1% versus 42.6%, OR = 0.63, 95% CI 0.51 to 0.78; p < 0.0001) (χ^2^ = 7.75, p = 0.1, I^2^ = 48%).

#### Neural invasion

Six studies (1, 4–6, 8, 14) were incorporated, and a significantly higher incidence of peri-neural infiltration in patients with PDCA was acquired (65.4% versus 77.0%, OR = 0.47, 95% CI 0.28 to 0.79; p = 0.005) (χ^2^ = 33.49, p < 0.00001, I^2^ = 85%) (**Figure 2F**). Heterogeneity analysis indicated the absence of a remarkable source of heterogeneity.

#### Tumor size

Six studies (1, 4, 6, 8, 12, 14) were incorporated, and the pooled result revealed that patients with PDCA had a significantly larger tumor size than patients with DCCA (weighted mean difference (WMD) = −0.55; 95% CI −0.72 to −0.38; p < 0.00001) (χ^2^ = 15.38, p = 0.009, I^2^ = 67%) (**Figure 2G**). Heterogeneity analysis indicated the absence of a remarkable source of heterogeneity.

#### Morbidities

Five studies (1, 4, 5, 8, 12) were incorporated, and no significant difference was acquired (53.8% versus 46.2%, OR = 1.43, 95% CI 0.77 to 2.64; p = 0.26) (**Figure 2H**). High heterogeneity (χ^2^ = 13.61, p = 0.009, I^2^ = 71%) was detected, and heterogeneity analysis indicated that when the study by Hatzaras et al. (1) was removed, a lower heterogeneity with a significantly higher incidence of postoperative morbidities in patients with DCCA was acquired (57.4% versus 47.3%, OR = 1.80, 95% CI 1.10 to 2.93; p = 0.02) (χ^2^ = 6.42, p = 0.09, I^2^ = 53%).

#### Postoperative biliary fistula

Three studies (4, 5, 8) were incorporated, and the pooled result revealed no significant difference between the two groups (5.7% versus 4.4%, OR = 1.30, 95% CI 0.62 to 2.72; p = 0.49) (χ^2^ = 2.27, p = 0.32, I^2^ = 12%) (**Figure 2I**).

#### Postoperative pancreatic fistula (Clavien grade B to C)

Three studies (4, 5, 8) were incorporated, and a significantly higher incidence of pancreatic fistula in patients with DCCA was acquired (41.8% versus 15.7%, OR = 3.52, 95% CI 2.46 to 5.06; p < 0.00001) (χ^2^ = 2.32, p = 0.31, I^2^ = 14%) (**Figure 2J**).

#### Mortalities

Five studies (1, 4, 5, 8, 12) were incorporated, and no significant difference was detected (2.9% versus 2.5%, OR = 1.22, 95% CI 0.51 to 2.92; p = 0.65) (χ^2^ = 2.20, p = 0.70, I^2^ = 0%) (**Figure 2K**).

#### Overall survival

Nine studies (1, 4, 7–9, 12–15) were incorporated, and a significantly better OS was acquired in patients with DCCA (hazard ratio (HR) = 0.81, 95% CI 0.70 to 0.95, p = 0.009) (χ^2^ = 30.93, p = 0.0001, I^2^ = 74%) (**Figure 2L**). Significant heterogeneity was detected, and heterogeneity analysis indicated that when the study by Hatzaras I et al. (1) was removed, patients with DCCA had a much better prognosis than patients with PDCA (HR = 0.77, 95% CI 0.74 to 0.80, p < 0.00001) (χ^2^ = 13.84, p = 0.05, I^2^ = 49%).

#### Disease-free survival

Three studies (5, 6, 8) with four outcomes (two outcomes from the study by Andrianello et al.: with and without adjuvant therapies) were incorporated, and no significant difference was acquired (HR = 1.20, 95% CI 0.68 to 2.12, p = 0.52) (χ^2^ = 17.81, p = 0.0005, I^2^ = 83%) (**Figure 2M**). However, when the study by Ethun et al. was removed, low heterogeneity with a significantly better DFS was achieved in patients with DCCA (HR = 1.69, 95% CI 1.15 to 2.20, p = 0.005) (χ^2^ = 0.26, p = 0.88, I^2^ = 0%).

### Publication bias, sensitivity analysis, and heterogeneity analysis

As shown in **Table 2**, among all the comparisons except for mortalities, the p-values in Egger’s test were all higher than 0.05, indicating the absence of remarkable bias. As for the comparison of postoperative mortalities, the p-value of Egger’s test was <0.05 (p = 0.011). Begg’s funnel plot and filled funnel plot (meta-trim command) were used for further evaluation (**Figure S1**). The result after trimming was similar to the result before trimming, indicating the absence of remarkable bias. The results of sensitivity analyses and heterogeneity analyses are recorded in the *Results* section of our manuscript.

## Discussion

DCCA and PDCA have similar malignancies, sharing the common pancreatico-biliary epithelium. Owing to the rarity of DCCA, especially in western countries, little has been known about the similarities and differences between DCCA and PDCA. Previous studies have indicated that DCCA and PDCA shared similar tumor biological features (17, 18). However, the World Health Organization (WHO) has indicated that DCCA and PDCA are two independent entities (19). Therefore, we performed the current meta-analysis to systematically evaluate the consistencies and inconsistencies of these two rare entities. Our major findings are as follows:

1. Obstructive jaundice and subsequent preoperative stenting are more frequently detected in patients with DCCA.
2. Patients with PDCA tend to have a larger tumor size, and PDCA exhibited more aggressively, with node metastasis and neural invasion more frequently detected in patients with PDCA. Patients with DCCA had a significantly higher R0 resection rate.
3. After curative-intent resection, patients with DCCA have more morbidities, and the incidence of POPF is significantly higher in patients with DCCA.
4. Patients with DCCA had a more favorable prognosis than patients with PDCA.

### Diagnostic challenge

To accurately distinguish DCCA from PDCA preoperatively is rather technically challenging and confusing. Regarding preoperative laboratory examinations, such as tumor biomarker CA199, previous observations often showed no meaningful results (1). As for radiological approaches, such as computed tomography (CT) and magnetic resonance imaging, these modalities can contribute to confirming the site of the tumor origin of intra-pancreatic lesions to some extent. However, regarding lesions within the pancreatic head, the correct localization could be more difficult (20). Both tumors often present as a solitary mass, adjacent to the ampullary or within the pancreas in the imaging of CT scan or ultrasound. Moreover, an endoscopic exploration often failed to provide valuable information, for they are both adenocarcinomas (21). Hence, accurate diagnosis mainly relies on intra- or postoperative pathological specimen evaluation. Macroscopically, the location of the tumor epicenter may contribute to a better distinction because DCCA mainly arises from the bile duct wall, until the posterior-cranial aspect of the pancreatic head, above or at the level of the ampulla (4). The cancer tissue of DCCA often spreads in a circumferential manner and therefore forms a constrictive lesion along the bile duct. Obstructive jaundice due to tumor infiltration often occurs earlier and is rather severe, which has been validated in our study that the proportion of patients with preoperative jaundice is extremely higher in patients with DCCA (p = 0.0003). As was acquired in our analysis, the tumor size of patients with DCCA was often smaller than that of patients with PDCA (p < 0.00001). A smaller tumor size in DCCA would cause earlier-period jaundice and would lead to an early diagnosis. In contrast to DCCA, pancreatic tumors can be found in any part of the pancreas, and the obstructive is often much later due to a delayed infiltration of the common bile duct. It is also unusual for PDCA to involve the distal bile duct circumferentially in a rather disorganized manner (13). Microscopically, there is no valid evidence suggesting the accurate differentiation of DCCA and PDCA, except for cases with precursor lesions (bile ductal or pancreatic) identified. It is worth mentioning that there are no specific immune-histochemical markers for distinguishing DCCA from PDCA (4). Additionally, although the ultrasound-guided invasive tissue biopsy may provide pathological confirmation, the risk of tumor dissemination, hemorrhage, organ injuries, or inflammation has made this medical procedure more technically challenging and less widely applicable (22–24). These factors all make the precise diagnosis of DCCA and PDCA more confusing. However, recently, promising results were reported by Gkolfakis et al.; in their study, the results of a network meta-analysis indicated that 22-gauge size end-cutting fine-needle biopsy needles showed the most favorable diagnostic performance for pancreatic masses with an extremely low false-negative rate (25). This unexpected finding may help clinicians better distinguish DCCA from PDCA.

### Surgical and pathological findings

In our meta-analysis, a total of 2,261 patients with DCCA and 29,996 patients with PDCA received curative-intent PD. Pooled results revealed that patients with DCCA had a significantly higher R0 resection rate than patients with PDCA (p < 0.0001). A similar result was also reported by other authors (4, 6). Further exploring its potential reasons, we accounted for it for the following reasons. First, due to the tumor location of DCCA, that is, its location is often within the bile duct lumen, DCCA tends to cause symptomatic obstructive jaundice earlier with a less advanced stage and a smaller tumor size at the time of diagnosis as well as patients receiving curative surgery. Numerous studies have also reported that patients with DCCA might present with more symptomatic pancreatic symptoms, including pain, abdominal fullness, early satiety, and weight loss (26, 27). Second, previous studies have proved that patients with PDCA had a significantly higher incidence of portal vein reconstruction (4, 6). Moreover, lymph node metastasis (p < 0.00001) and neural invasion (p = 0.005) were more frequently detected in patients with PDCA, reflecting the fact that the majority of patients with PDCA were diagnosed in a more advanced stage, and therefore, achieving a negative margin could be more difficult. Our meta-analysis revealed a significantly lower R0 rate in patients with PDCA (p < 0.0001). However, conversely, the study by Garnier et al. (4) reported a similar R0 resection rate among patients with DCCA and PDCA. Garnier et al. analyzed its potentially reasonable reasons and accounted for the modern cohort (2010–2018) of their study. Compared with the study by Ethun et al. (6) (2000–2015), the great evolvement in PD specimen analyses in the late 2000s, that is, the highlight of the venous groove invasion in patients with DCCA since then, would increase the R1 rate of DCCA (28). Additionally, in the study by Garnier et al., the application of neo-adjuvant therapies in patients with PDCA might also cause a lower R1 rate (4). After neo-adjuvant therapies, residual cancer often consists of scattered tumor foci separated by stretches of non-neoplastic tissue, which would lead to a higher possibility of negative margins (29). In short, based on our findings as well as the observations reported by others, PDCA tends to be more advanced with a lower R0 resection rate and higher incidences of node metastasis and neural invasion. Evaluating the surgical margins can be problematic after the application of neo-adjuvant therapies, and future well-designed studies are required for further exploration.

### Morbidities

After the application of curative PD, patients with DCCA had a significantly higher incidence of postoperative complications, especially the incidence of POPF (Clavien grade B to C) (p < 0.00001) and other pancreas-associated complications. Our findings were consistent with the observations reported in previously published literature (16, 30, 31). Based on the International Study Group on Pancreatic Fistula, pancreatic duct size <3 mm and soft pancreatic parenchyma are all risk factors for POPF (32). As was acquired in our analysis, patients with DCCA had a smaller tumor size than patients with PDCA (p < 0.00001). The DCCA would mainly infiltrate the main bile duct rather than cause obstructive pancreatitis. An un-dilated pancreatic duct and the soft pancreatic parenchyma without tumor infiltration would greatly increase the risk of POPF. One previous study has demonstrated that a hard pancreas is advantageous for surgeons because the infiltration of the pancreatic duct seemed to have increased the mechanical strength, leading to a more solid pancreaticojejunostomy (33). Consequently, the earlier diagnosis of DCCA tends to cause more severe post-anastomosis pancreatic fistula, linked with postoperative mortalities, various complications (delayed gastric emptying), and a longer postoperative hospital stay (34).

### Prognosis

The prognosis of patients with DCCA is significantly better than that of patients with PDCA in our analysis (p < 0.00001), which is in line with the observations reported by others (4–6, 9, 35). The most likely explanation for this phenomenon is that patients with PDCA were more frequently diagnosed in an advanced stage with delayed-observed obstructive jaundice. The incidences of node metastasis and neural invasion were significantly higher in patients with PDCA. Node metastasis and neural invasion have both been demonstrated as independent prognostic factors for peri-ampullary cancers (1). With regard to patients with DCCA, earlier-detected obstructive jaundice would introduce an earlier diagnosis as well as more timely curative surgery. Consequently, a significantly higher R0 rate could be detected more frequently in patients with DCCA. One previous meta-analysis with 2,063 patients with DCCA included has indicated that peri-neural invasion, R0 resection rate, and node metastasis were all independent prognostic factors (36). Lymph node status, margin status, and neural invasion have also been regarded as the prognostic factors for peri-ampullary cancers in general (37, 38). Interestingly, in the study by Guilbaud et al., after controlling the factors margin status, node metastasis, and tumor size *via* propensity score matching analysis, patients with DCCA had a similar prognosis versus patients with PDCA (8), which further validated the fact that tumor biology seemed to have the strongest weight of evidence of predicting survival (39–41). Moreover, the earlier diagnosis of DCCA with less advanced disease often leads to a higher R0 rate, which further promotes a better prognosis of DCCA.

There are several limitations to our study. First, the retrospective nature of the majority of included studies would introduce bias. Second, owing to the rarity of DCCA, especially in western countries, the comparison between DCCA and PDCA would be less convincing. Third, the estimation of HRs *via* Tierney’s method might introduce bias. Fourth, the deficiency of the original date also hindered deeper exploration.

## Conclusion

DCCA had more favorable tumor pathological features and prognosis than PDCA, and preoperative jaundice was more common in patients with DCCA. Moreover, even after the same surgical procedure, PD, a significantly higher incidence of postoperative complications, especially POPF, was more common in patients with DCCA.

## Author contributions

T-RL and J-MW contributed equally to the study. T-RL contributed to data acquisition and drafted the manuscript. W-JM, Y-FH, Y-SD and Y-WJ were involved in editing the manuscript. F-YL contributed to the study design and revision of the manuscript. All authors contributed to the article and approved the submitted version.

## Supplementary material

Supplementary Figure 1 Further evaluation on the potential publication bias within the comparison of Mortalities. **(A)** Begg’s funnel plot. **(B)** Filled funnel plot (meta-trim command).

## References

[1] Hatzaras I George N Muscarella P Melvin WS Ellison EC Bloomston M . Predictors of survival in periampullary cancers following pancreaticoduodenectomy. Ann Surg Oncol (2010) 17(4):991–7. doi: 10.1245/s10434-009-0883-9 PMC2861840 20108122

[2] Cameron JL He J . Two thousand consecutive pancreaticoduodenectomies. J Am Coll Surg (2015) 220(4):530–6. doi: 10.1016/j.jamcollsurg.2014.12.031 25724606

[3] Siegel RL Miller KD Jemal A . Cancer statistics, 2016. CA Cancer J Clin (2016) 66(1):7–30. doi: 10.3322/caac.21332 26742998

[4] Garnier J Ewald J Poizat F Traversari E Marchese U Palen A . Prospective evaluation of resection margins using standardized specimen protocol analysis among patients with distal cholangiocarcinoma and pancreatic ductal adenocarcinoma. J Clin Med (2021) 10(15):3247. doi: 10.3390/jcm10153247 PMC8348230 34362031

[5] Andrianello S Marchegiani G Malleo G Rusev BC Scarpa A Bonamini D . Over 700 whipples for pancreaticobiliary malignancies: Postoperative morbidity is an additional negative prognostic factor for distal bile duct cancer. J Gastrointest Surg (2017) 21(3):527–33. doi: 10.1007/s11605-016-3328-3 27882511

[6] Ethun CG Lopez-Aguiar AG Pawlik TM Poultsides G Idrees K Fields RC . Distal cholangiocarcinoma and pancreas adenocarcinoma: Are they really the same disease? a 13-institution study from the US extrahepatic biliary malignancy consortium and the central pancreas consortium. J Am Coll Surg (2017) 224(4):406–13. doi: 10.1016/j.jamcollsurg.2016.12.006 PMC10191774 28017812

[7] Hester CA Dogeas E Augustine MM Mansour JC Polanco PM Porembka MR . Incidence and comparative outcomes of periampullary cancer: A population-based analysis demonstrating improved outcomes and increased use of adjuvant therapy from 2004 to 2012. J Surg Oncol (2019) 119(3):303–17. doi: 10.1002/jso.25336 30561818

[8] Guilbaud T Girard E Lemoine C Schlienger G Alao O Risse O . Intra-pancreatic distal cholangiocarcinoma and pancreatic ductal adenocarcinoma: A common short and long-term prognosis? Updates Surg (2021) 73(2):439–50. doi: 10.1007/s13304-021-00981-0 33486711

[9] Muttillo EM Ciardi A Saullo P Troiano R Masselli G Guida M . A prognostic score for predicting survival in patients with pancreatic head adenocarcinoma and distal cholangiocarcinoma. In Vivo (Athens Greece) (2021) 35(1):507–15. doi: 10.21873/invivo.12285 PMC7880773 33402503

[10] Liberati A Altman DG Tetzlaff J Mulrow C Gotzsche PC Ioannidis JP . The PRISMA statement for reporting systematic reviews and meta-analyses of studies that evaluate health care interventions: Explanation and elaboration. PloS Med (2009) 6(7):e1000100. doi: 10.1371/journal.pmed.1000100 19621070 PMC2707010

[11] Lv TR Liu F Hu HJ Regmi P Ma WJ Yang Q . The role of extra-hepatic bile duct resection in the surgical management of gallbladder carcinoma. a first meta-analysis. Eur J Surg Oncol (2022) 48(3):482–91. doi: 10.1016/j.ejso.2021.11.131 34955314

[12] van Geenen RC van Gulik TM Offerhaus GJ de Wit LT Busch OR Obertop H . Survival after pancreaticoduodenectomy for periampullary adenocarcinoma: An update. Eur J Surg Oncol (2001) 27(6):549–57. doi: 10.1053/ejso.2001.1162 11520088

[13] Gonzalez RS Bagci P Basturk O Reid MD Balci S Knight JH . Intrapancreatic distal common bile duct carcinoma: Analysis, staging considerations, and comparison with pancreatic ductal and ampullary adenocarcinomas. Modern Pathol (2016) 29(11):1358–69. doi: 10.1038/modpathol.2016.125 PMC5598556 27469329

[14] He J Ahuja N Makary MA Cameron JL Eckhauser FE Choti MA . 2564 Resected periampullary adenocarcinomas at a single institution: Trends over three decades. HPB (Oxford) (2014) 16(1):83–90. doi: 10.1111/hpb.12078 23472829 PMC3892319

[15] Riall TS Cameron JL Lillemoe KD Winter JM Campbell KA Hruban RH . Resected periampullary adenocarcinoma: 5-year survivors and their 6- to 10-year follow-up. Surgery (2006) 140(5):764–72. doi: 10.1016/j.surg.2006.04.006 17084719

[16] Yeo CJ Cameron JL Sohn TA Lillemoe KD Pitt HA Talamini MA . Six hundred fifty consecutive pancreaticoduodenectomies in the 1990s: Pathology, complications, and outcomes. Ann Surg (1997) 226(3):248–57; discussion 57-60. doi: 10.1097/00000658-199709000-00004 9339931 PMC1191017

[17] Bledsoe JR Shinagare SA Deshpande V . Difficult diagnostic problems in pancreatobiliary neoplasia. Arch Pathol Lab Med (2015) 139(7):848–57. doi: 10.5858/arpa.2014-0205-RA 26125425

[18] Nakanuma Y Sato Y . Hilar cholangiocarcinoma is pathologically similar to pancreatic duct adenocarcinoma: Suggestions of similar background and development. J Hepatobiliary Pancreat Sci (2014) 21(7):441–7. doi: 10.1002/jhbp.70 24446421

[19] Nagtegaal ID Odze RD Klimstra D Paradis V Rugge M Schirmacher P . The 2019 WHO classification of tumours of the digestive system. Histopathology (2020) 76(2):182–8. doi: 10.1111/his.13975 PMC7003895 31433515

[20] Mangiavillano B Mariani AA Petrone MC . An intrapancreatic cholangiocarcinoma detected with optical coherence tomography during endoscopic retrograde cholangiopancreatography. Clin Gastroenterol Hepatol (2008) 6(6):A30. doi: 10.1016/j.cgh.2008.02.004 18407794

[21] Dumonceau JM Polkowski M Larghi A Vilmann P Giovannini M Frossard JL . Indications, results, and clinical impact of endoscopic ultrasound (EUS)-guided sampling in gastroenterology: European society of gastrointestinal endoscopy (ESGE) clinical guideline. Endoscopy (2011) 43(10):897–912. doi: 10.1055/s-0030-1256754 21842456

[22] Lewis AR Valle JW McNamara MG . Pancreatic cancer: Are "liquid biopsies" ready for prime-time? World J Gastroenterol (2016) 22(32):7175–85. doi: 10.3748/wjg.v22.i32.7175 PMC4997639 27621566

[23] Fujii LL Levy MJ . Basic techniques in endoscopic ultrasound-guided fine needle aspiration for solid lesions: Adverse events and avoiding them. Endoscopic Ultrasound (2014) 3(1):35–45. doi: 10.4103/2303-9027.123006 24949409 PMC4063261

[24] Rimbas M Deaconu M Croitoru A Haidar A . Sudden appearance of free fluid during endoscopic ultrasound-guided fine-needle aspiration. Endoscopic Ultrasound (2016) 5(1):55–7. doi: 10.4103/2303-9027.175900 PMC4770624 26879168

[25] Gkolfakis P Crinò SF Tziatzios G Ramai D Papaefthymiou A Papanikolaou IS . Comparative diagnostic performance of end-cutting fine-needle biopsy needles for EUS tissue sampling of solid pancreatic masses: A network meta-analysis. Gastrointest Endosc (2022) 95(6):1067–77.e15. doi: 10.1016/j.gie.2022.01.019 35124072

[26] Veillette G Castillo CF . Distal biliary malignancy. Surg Clinics North America (2008) 88(6):1429–47. doi: 10.1016/j.suc.2008.07.003 18992603

[27] DiMagno EP . Pancreatic cancer: Clinical presentation, pitfalls and early clues. Ann Oncol (1999) 10 Suppl 4:140–2. doi: 10.1093/annonc/10.suppl_4.S140 10436807

[28] Kamposioras K Anthoney A Fernández Moro C Cairns A Smith AM Liaskos C . Impact of intrapancreatic or extrapancreatic bile duct involvement on survival following pancreatoduodenectomy for common bile duct cancer. Br J Surg (2014) 101(2):89–99. doi: 10.1002/bjs.9367 24375301

[29] Kleive D Labori KJ Line PD Gladhaug IP Verbeke CS . Pancreatoduodenectomy with venous resection for ductal adenocarcinoma rarely achieves complete (R0) resection. HPB (Oxford) (2020) 22(1):50–7. doi: 10.1016/j.hpb.2019.05.005 31186199

[30] Vollmer CM Jr. Sanchez N Gondek S McAuliffe J Kent TS Christein JD . A root-cause analysis of mortality following major pancreatectomy. J Gastrointest Surg (2012) 16(1):89–102; discussion -3. doi: 10.1007/s11605-011-1753-x 22065319

[31] Čečka F Jon B Šubrt Z Ferko A . Clinical and economic consequences of pancreatic fistula after elective pancreatic resection. Hepatobiliary Pancreatic Dis Int (2013) 12(5):533–9. doi: 10.1016/s1499-3872(13)60084-3 24103285

[32] Pratt WB Callery MP Vollmer CM Jr . Risk prediction for development of pancreatic fistula using the ISGPF classification scheme. World J Surg (2008) 32(3):419–28. doi: 10.1007/s00268-007-9388-5 18175170

[33] Belyaev O Rosenkranz S Munding J Herzog T Chromik AM Tannapfel A . Quantitative assessment and determinants of suture-holding capacity of human pancreas. J Surg Res (2013) 184(2):807–12. doi: 10.1016/j.jss.2013.04.017 23663821

[34] Callery MP Pratt WB Kent TS Chaikof EL Vollmer CM Jr . A prospectively validated clinical risk score accurately predicts pancreatic fistula after pancreatoduodenectomy. J Am Coll Surg (2013) 216(1):1–14. doi: 10.1016/j.jamcollsurg.2012.09.002 23122535

[35] Oh TG Chung MJ Bang S Park SW Chung JB Song SY . Comparison of the sixth and seventh editions of the AJCC TNM classification for gallbladder cancer. J Gastrointest Surg (2013) 17(5):925–30. doi: 10.1007/s11605-012-2134-9 23299221

[36] Wellner UF Shen Y Keck T Jin W Xu Z . The survival outcome and prognostic factors for distal cholangiocarcinoma following surgical resection: A meta-analysis for the 5-year survival. Surg Today (2017) 47(3):271–9. doi: 10.1007/s00595-016-1362-0 27236779

[37] Bergeat D Turrini O Courtin-Tanguy L Truant S Darnis B Delpero JR . Impact of adjuvant chemotherapy after pancreaticoduodenectomy for distal cholangiocarcinoma: A propensity score analysis from a French multicentric cohort. Langenbecks Arch Surg (2018) 403(6):701–9. doi: 10.1007/s00423-018-1702-1 30112638

[38] Jun SY Sung YN Lee JH Park KM Lee YJ Hong SM . Validation of the eighth American joint committee on cancer staging system for distal bile duct carcinoma. Cancer Res Treat (2019) 51(1):98–111. doi: 10.4143/crt.2017.595 29510611 PMC6333967

[39] Cameron JL Crist DW Sitzmann JV Hruban RH Boitnott JK Seidler AJ . Factors influencing survival after pancreaticoduodenectomy for pancreatic cancer. Am J Surg (1991) 161(1):120–4; discussion 4-5. doi: 10.1016/0002-9610(91)90371-J 1987845

[40] Yeo CJ Sohn TA Cameron JL Hruban RH Lillemoe KD Pitt HA . Periampullary adenocarcinoma: Analysis of 5-year survivors. Ann Surg (1998) 227(6):821–31. doi: 10.1097/00000658-199806000-00005 PMC1191384 9637545

[41] Bassi C Dervenis C Butturini G Fingerhut A Yeo C Izbicki J . Postoperative pancreatic fistula: An international study group (ISGPF) definition. Surgery (2005) 138(1):8–13. doi: 10.1016/j.surg.2005.05.001 16003309

