# Supplementary figures and images for: The consistencies and inconsistencies between distal cholangiocarcinoma and pancreatic ductal adenocarcinoma: A systematic review and meta-analysis
Source: Front Oncol. 2022 Dec 12;12:1042493. doi: 10.3389/fonc.2022.1042493 (PMC9791204; doi:10.3389/fonc.2022.1042493)

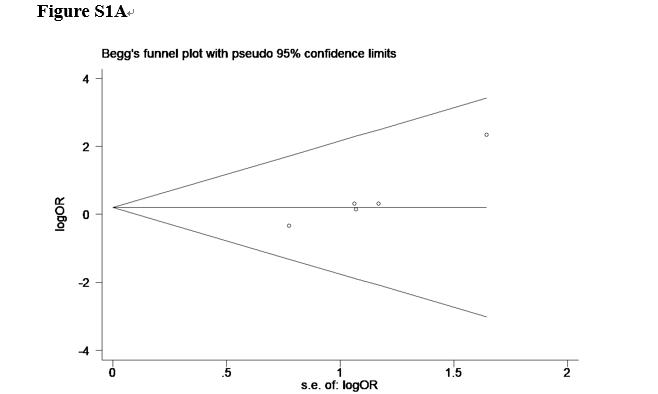

Supplement: Supplementary Figure 1 — Further evaluation on the potential publication bias within the comparison of Mortalities. (A) Begg’s funnel plot. (B) Filled funnel plot (meta-trim command). [file Image_1.jpeg]

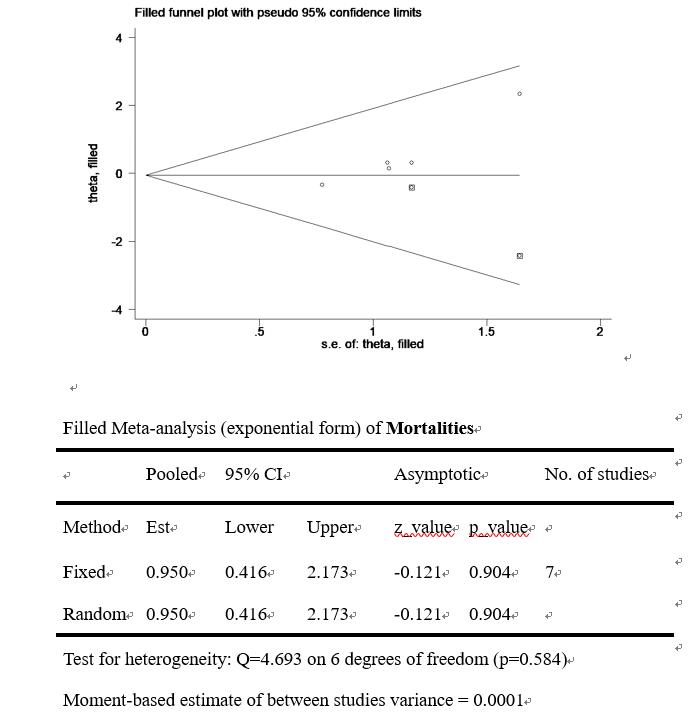

Supplement: Supplementary file 2 [file Image_2.jpeg]
